# PI3Kδ Inhibitors as Immunomodulatory Agents for the Treatment of Lymphoma Patients

**DOI:** 10.3390/cancers13215535

**Published:** 2021-11-04

**Authors:** Chiara Tarantelli, Lisa Argnani, Pier Luigi Zinzani, Francesco Bertoni

**Affiliations:** 1 Institute of Oncology Research, Faculty of Biomedical Sciences, USI, 6500 Bellinzona, Switzerland; chiara.tarantelli@ior.usi.ch; 2 IRCCS Azienda Ospedaliero-Universitaria di Bologna, 40138 Bologna, Italy; lisa.argnani@unibo.it (L.A.); pierluigi.zinzani@unibo.it (P.L.Z.); 3 Istituto di Ematologia “Seràgnoli”, Dipartimento di Medicina Specialistica, Diagnostica e Sperimentale, Università degli Studi di Bologna, 40138 Bologna, Italy; 4 Oncology Institute of Southern Switzerland, Ente Ospedaliero Cantonale, 6500 Bellinzona, Switzerland

**Keywords:** lymphoma, PI3K inhibitors, T-cells, B-cells, macrophages, chemokine, cancer, tumor, immune checkpoint inhibitors

## Abstract

**Simple Summary:**

This review focuses on the effects that a class of drugs, PI3Kδ inhibitors, used for the treatment of patients with lymphoma can have not on the neoplastic cells but on the normal cells and how this effect can modulate the immune response and potentially contribute to the anti-tumor response.

**Abstract:**

The development of small molecules able to block specific or multiple isoforms of phosphoinositide 3-kinases (PI3K) has already been an active field of research for many years in the cancer field. PI3Kδ inhibitors are among the targeted agents most extensively studied for the treatment of lymphoma patients and PI3Kδ inhibitors are already approved by regulatory agencies. More recently, it became clear that the anti-tumor activity of PI3K inhibitors might not be due only to a direct effect on the cancer cells but it can also be mediated via inhibition of the kinases in non-neoplastic cells present in the tumor microenvironment. T-cells represent an important component of the tumor microenvironment and they comprise different subpopulations that can have both anti- and pro-tumor effects. In this review article, we discuss the effects that PI3Kδ inhibitors exert on the immune system with a particular focus on the T-cell compartment.

## 1. Introduction

Phosphoinositide 3-kinases (PI3Ks) are a class of enzymes fundamental in the regulation of cell metabolism, proliferation and survival [1,2,3,4,5,6]. PI3Ks are active in most human cancers, often representing oncogenic drivers due to genetic events directly targeting their coding genes or determining the constitutive activation or upstream components of the signaling cascade [1,4,5,6].

PI3Ks comprise four isoforms p110α (PI3Kα, coded by the *PIK3CA* gene), p110β (PI3Kβ, coded by *PIK3CB*), p110δ (PI3Kδ, coded by *PIK3CD*), and p110γ (PI3Kγ, coded by *PIK3CG*). These are the catalytic subunits that form heterodimers with regulatory isoforms. The p85α and its splicing variants p55α and p50α (*PIK3R1*), p85β (*PIK3R2*), and p55γ (*PIK3R3*) can bind PI3Kα, PI3Kβ, or PI3Kδ (class IA PI3Ks), while p101 (*PIK3R5*) or p87 (*PIK3R6)* bind PI3Kγ (Class IB PI3Ks). PI3Kδ and PI3Kγ are largely restricted to leukocytes, while PI3Kα and PI3Kβ are ubiquitously expressed [1,4,5,6].

The development of small molecules able to block specific or multiple PI3K isoforms is a heavily pursued effort in oncology: Table 1 shows the PI3Kδ inhibitors that have entered clinical development. Multiple preclinical and clinical studies showed the anti-tumor activity of PI3Kδ inhibitors in patients affected by chronic lymphocytic leukemia (CLL), B- and T-cell lymphomas and these data have been extensively summarized elsewhere [3,4,5,6,7,8]. Importantly, PI3Ks are expressed not only in the cancer cells, but also in the non-neoplastic cells, in which PI3K inhibitors contribute to their pro- or anti-tumor effects, and they can be used to improve the response to immunomodulatory and immunotherapeutic agents [9,10,11,12,13,14,15,16,17]. In this review article, we will discuss the effects of inhibiting PI3Kδ isoform on the immune system with a particular focus on the T-cell compartment.

## 2. Immune System and Anti-Cancer Immunotherapy

The TME is a complex system comprising the cancer cells, plus proteins and other chemical components of the extracellular matrix (ECM), and “accessory” non-neoplastic cells, such as resident mesenchymal support cells, infiltrating inflammatory immune cells, and endothelial cells. Altogether, the tumor microenvironment (TME) plays a fundamental role in regulating tumor development, both leading to an immune inflammatory response and fueling innate and adaptive immune activity against cancer cells, but also supporting the growth of the latter [50].

Cells and tissues are continuously surveilled by immune cells, which recognize and eliminate emerging cancer cells. Genetically engineered mice deficient for CD8+ cytotoxic T-lymphocyte (CTLs), CD4+ Th1 helper T-cells, or natural killer (NK) cells components of the immune system, show an increased tumor incidence [51,52].

During tumor initiation, naïve T-cells recognize antigens expressed by malignant cells are primed in the draining lymph nodes, are activated, and migrate in the TME. In this niche, immune response eliminates immunogenic cancer cells [53]. NK and cytotoxic CD8+ T-cells eliminate immunogenic proliferating cancer cells [54]. Later on, inflammation is persistent and inflammatory cells are recruited and activated. In many cancers, high presence of tumor-infiltrated T-cells has a good prognostic value [55,56]; instead, high presence of macrophage infiltration often correlates with poor prognosis [57] and tumor-associated inflammatory response has a paradoxical effect of enhancing tumor progression [58,59]. Tumor-promoting effects of immune cells is becoming more and more evident and inflammation provides bioactive factors that helps proliferative growth, angiogenesis, invasion, and metastasis. In the setting of T-cell lymphomas, expression and secretion of immunoinhibitory molecules can be shared by both tumor and non-neoplastic cells [15,17].

CD8+ T-cells are the main players among anti-tumor T-cells. They are primed and activated by antigen presenting cells (APC) to differentiate into CTL, which can directly kill cancer cells [60].

CD4+ T helper 1 (Th-1) cells act through a variety of mechanisms. They massively secrete proinflammatory cytokines, as IL-2, TNF-α and IFN-γ, co-adjuvate CTL cytotoxicity and T-cell priming and activation, help macrophages and NK cells to destroy tumoral cells and facilitate tumor antigens presentation [61,62,63]. Immune infiltrate components of tumors include CD8+ T-cells and Th-1 cytokines, correlating with favorable prognosis in many cancer types [64].

Effector T-cells killing-activity relies on the balance between the capability of tumor antigens to induce an immune response (immunogenic feature) and the existence of signals impairing T-cell functions [53]. This process by which the immune system controls tumoral growth and balances tumor immunogenicity is called immune editing. Tumoral cells with the most immunogenic antigens are recognized and killed in the early stages of tumorigenesis, while the less immunogenic cancer cells escape T-cell control [54,65]. Neoplastic cells are also able to decrease the response of the others innate immunity involved cells as tumor-associated macrophages (TAM) and NK cells [66].

At present, different mechanisms of cancer immune tolerance have been identified. Immune checkpoints signals are negative regulators of effector T-cells, and the two mainly studied molecules in cancers are Cytotoxic T-Lymphocyte Associated Protein 4 (CTLA-4) and Programmed Cell Death 1 (PD-1) [63]. Known ligands of CTLA-4 are CD80 and CD86, while PD-1 binds to its coreceptors PDL-1/2, expressed also by cancer cells, to impair anti-tumor T-cell responses [67]. Immune checkpoint inhibitors (pembrolizumab and nivolumab as anti-PD1; atezolizumab as anti-PD-L1; ipilimumab as anti-CTLA4) became a successful strategy to enhance anti-tumor response in many malignancies [68].

**Figure 1:**
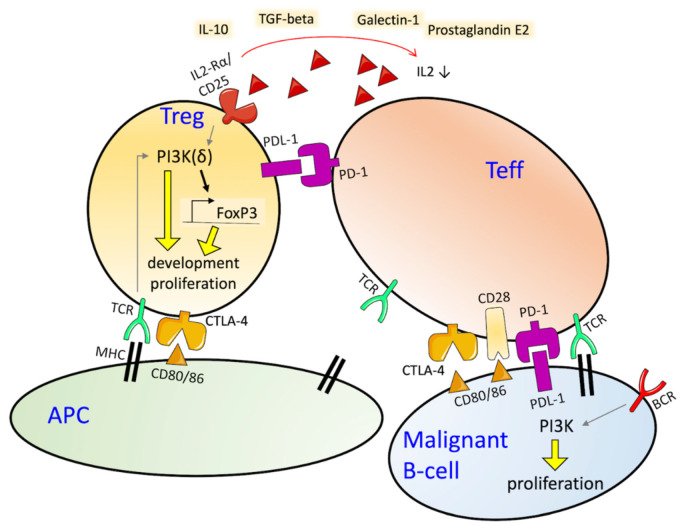
Immuno-regulation in the tumor microenvironment. Tregs deprive the surrounding of co-stimulatory signals for effector T-cells (Teff) affinity and activity. They exert their suppressive mechanism by different modalities: depriving IL-2 from the surrounding, therefore reducing it for effector T-cells, by IL-2 binding with CD25; constitutively expressing CTLA-4, which down-regulates CD80/86 expression by antigen-presenting cells (APC) and limits co-stimulatory signals for Teff, together with CD28; immune-suppressive cytokines produced by Tregs decrease APC and Teff signals; PD-1/PD-L1 axis activation inhibits Teff function. In this environment, responders T-cells die by apoptosis or stay dormant and tumor cells are prone to proliferation. Targeting PI3K specifically in Tregs could provide advantage for anti-cancer immunotherapy. TCR: T-cell receptor; MHC: Major Histocompatibility Complex, BCR: B-cell receptor.

As the tumor grows, cancer cells and signal molecules in the TME recruit regulatory CD4+ T-cells (Tregs), responsible to inhibit T-cell responses, specifically priming, activation and cytotoxicity of effector immune cells (Figure 1) [69]. Tregs contribute to the suppression of uncontrolled clonal expansion and negatively regulate the insurgence of hyper-inflammatory state. In tumors, they are recruited to hamper the immune system and escape immune surveillance [70,71]. Tregs exploit their function through contact-dependent mechanisms—PDL-1, LAG-3 C39/73, CTLA-4 or PD1 are expressed on their cell surface, and lead to effector cell death or to enhance this event—and contact-independent mechanisms—by secreting immune-suppressive cytokines, as IL-10, TGF-β, prostaglandin E2, adenosine, and galectin-1 [72,73], and also recruiting myeloid-derived suppressor cells which contribute to build an immunosuppressive environment [74]. Tumor-infiltrating Treg cells are under pressure of a challenging environment with low oxygen availability, high glucose demand, and a multitude of cytokines and chemokines [75,76]. Tan et al. demonstrated that PI3K-AKT pathway regulates the immunosuppressive capacity of PD-1 deficient Tregs [77]. Tregs are characterized by the expression of the IL-2 receptor alpha chain (CD25), CD4, FOXP3 and CTLA-4. Enrichment of Tregs in tumors can be due to an augmented recruitment, expansion in the TME as a consequence of antigenic exposure, response to cytokine signals or metabolic changes (Figure 1) [53]. Higher numbers of Tregs have been detected in the blood of lymphoma patients than of healthy or cured patients [78,79], and in lymphoma tissues than in reactive lymph nodes [78]. In cutaneous diffuse large B-cell lymphoma, Hodgkin’s lymphoma and Epstein-Barr virus-associated lymphoma, Tregs are recruited by CCR4 ligands or evolve from conventional cells (Tconvs) to Tregs in the TME [80]. High presence of circulating Tregs represents a poor prognostic factor in diffuse large B-cell lymphoma (DLBCL), correlates with high lactate dehydrogenase, advanced stage of the disease [78], and poor survival [72,81].

## 3. Targeting PI3Kδ and Treg in Lymphomas

The ideal immune regulatory approach to fight cancer should be able to selectively deprive Tregs in TME, while maintaining a potent immune effector system. Understanding the signaling pathways regulating Tconvs and Tregs mechanisms could help to develop specific Tregs and Tconvs modulation strategies. In vitro and in vivo studies demonstrated that PI3K signaling is fundamental for Treg differentiation and immunosuppressive function, although the precise mechanism is still unclear [82,83,84].

**Figure 2:**
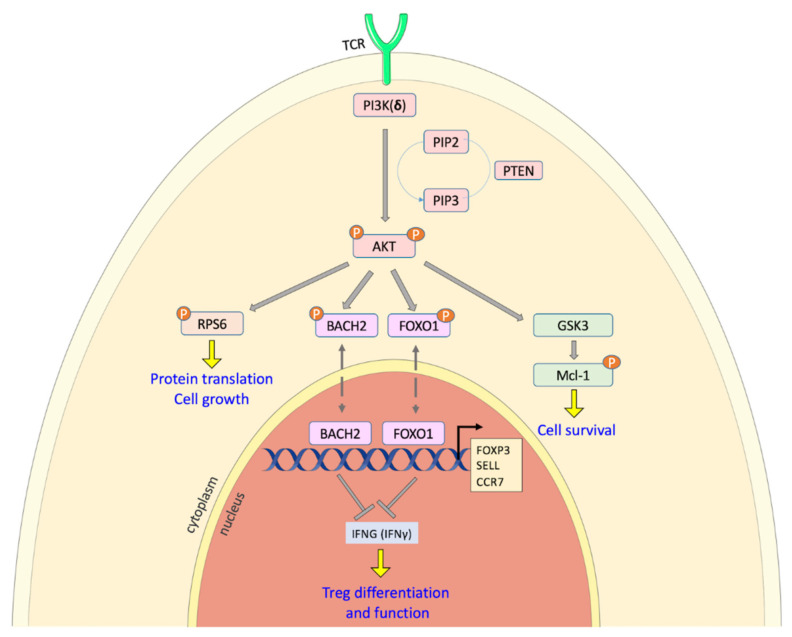
PI3K pathway in Tregs regulating anti-cancer immunity. Activated AKT pathway through PI3Kδ signaling, the dominant isoform in Tregs, leads to the phosphorylation of the BACH2 and FOXO1 transcription factors and in this form they are sequestrated in the cytoplasm. BACH2 and FOXO1 are regulators of genes involved in Treg differentiation and function, such as *FOXP3*, L-selectin (*SELL*), *CCR7* and *IFNγ*. PI3Kδ inhibition suppress Tregs functionality; they are not able to suppress any more anti-tumor responses. Proteins belonging to downstream TCR signaling are also regulated by PI3Kδ, as pS6 phosphorylation and GSK-3β activation, controlling proliferation, survival pathway and downstream degradation of the antiapoptotic protein Mcl-1.

Tregs are dependent on the activity of the PI3Kδ isoform [83,85,86] and studies suggest that loss of PI3K signaling in Tregs leads to increased activity of the BACH2 and FOXO1 transcription factors, which in normal conditions, regulate the expression of key genes in Treg differentiation and function (*FOXP3*, L-selectin, *CCR7* and *IFNγ*) [87,88,89] (Figure 2). In line with this mechanism, PTEN inhibition impairs Treg function and reduces their immunosuppression ability [90]. PTEN-deficient Tregs could reduce FOXO1 transcription, followed by decreased expression of FOXP3, essential for Treg development [91].

Interestingly, genetic and pharmacological inhibition of PI3Kδ in mice exerts anti-tumor activity via inhibition of Tregs and, possibly, of myeloid-derived suppressor cells [82,85]. In this context, CD8+ CTL can still mediate anti-tumor activity, although an altered balance between regulatory and effector CD4+ T-cells, with effector cells that prevail. Pharmacological targeting of PI3Kδ lead to similar changes compared to genetical inhibition, such as suppression of tumor growth and reduction of immunosuppression, in many cancer models [22,31,82,86,92,93,94,95,96]. The PI3Kδ inhibitor parsaclisib has in vivo antitumor activity against the A20 mouse lymphoma cell lines despite no in vitro anti-tumor activity [96]. Similar data are available for the PI3Kδ inhibitor linperlisib against models of breast carcinoma and colorectal cancer [22]. Hanna et al. demonstrated that PI3Kδ inhibition decreases Tregs numbers, proliferation, and activity in the Eμ-TCL1 model, but also CD8+ effector T-cells numbers and cytotoxicity T-cell ability [94]. In vitro experiments on T-cells from CLL patients, revealed that idelalisib down regulates the expression of crucial genes for T-cell mediated immunity, impairs IFNγ production by CD4 and CD8 T-cells, and decreases the proliferative capacity of T-cells without affecting their survival [93]. Similar results have been reported by Maharaj et al., also using the Eμ-TCL1 model [95]. The PI3Kδ inhibitor idelalisib and the PI3Kδ/γ inhibitor duvelisib, but not the dual PI3Kδ/CK1ε inhibitor umbralisib, determined a reduction of Tregs, which was associated with increased immune-mediated toxicities, in the absence of changes in the CD4/CD8 ratio or in the absolute number of T-cells [95]. In a syngeneic colorectal cancer model, treatment with the PI3Kδ inhibitor IOA-244 increases NK cells, and the ratio of cytotoxic CD8+ T-cells/Tregs [31]. The last observation is supported by data indicating a selective and concentration-dependent suppression of Treg cells but not of the proliferation of CD8+ T-cells [31]. Suppression of Tregs in syngeneic tumors is also reported with the PI3Kα/δ inhibitor copanlisib [92], and with KA2237 [38]. In an in vivo mammary tumor model, PI3Kδ blockade leads tumors to be divided in “non-regressors”, in which tumor growth rate is reduced but tumors continue to grow, and “regressors” where tumors shrink. Tumor infiltrating T-cells in “regressors” are enriched of elements indicating a CD8-specific T-cell response. In both groups of mice Tregs where reduced, although in Tregs from “non-regressor” tumors the expression of the coinhibitory receptor LAG3 is enriched compared to “regressor” and untreated tumors [97].

Exposure of follicular lymphoma (FL) cells, cocultured with follicular dendritic cells derived from normal tonsils, to idelalisib down-regulates the expression of integrins and their ligands, of proangiogenic factors and it determines a disruption of the CD40/CD40L-mediated crosstalk between FL cells and T-cells [98]. The PI3Kδ inhibitor down-regulates CCL22 expression, and this would reduce the recruitment of Tregs and of T follicular helper cells (TFH), both expressing the chemokine receptor CCR4 and supportive for the growth and survival of FL cells [98]. A similar effect is also observed during the generation of high-affinity antibodies in the GC, where PI3Kδ regulates TFH formation and function, activating ICOS, leading to intracellular signaling activation, production of TFH-related cytokines and effector molecules [99]. Moreover, idelalisib appears to increase the sensitivity of FL cells to the BCL2 inhibitor venetoclax, via a reduced PI3Kδ-mediated BAD phosphorylation, and/or via up-regulating the levels of proapoptotic factor HRK, and/or down-regulation of the anti-apoptotic factor BFL-1 [98].

Targeting PI3Kδ isoform with idelalisib stimulates CD8+ T-cells proliferation, maintaining survival, cytokines and granzyme B production. Idelalisib also inhibits Akt phosphorylation (both S473 and T308) in Tregs but not in Tconvs, and abrogates Tregs proliferation without affecting Tconv cells [83,86].

Finally, data collected in syngeneic mouse models mostly suggest that PI3Kδ inhibitors show synergism with immune checkpoint modulators [22,31,92]. However, there are also data demonstrating an important suppression of CD8+ T-cells maturation and killing capacity, antagonizing the effect due to immune checkpoint blockade [100].

## 4. Potential Toxicities Linked with PI3Kδ Inhibition in T-Cells

Side effects of PI3Kδ inhibitors encompass infections, hepatotoxicity, diarrhea and/or colitis, and pneumonitis [4,5,6,101,102,103,104,105,106] (Table 2). In clinical trials with idelalisib, serious adverse events have also included deaths related to cytomegalovirus infections, pneumonias caused by *Pneumocystis jirovencii*, in addition to respiratory events possibly caused by infections [107]. These toxicities have been linked with a T-cell immune response impairment induced by PI3Kδ inhibition that could favor such infections or viral reactivations, both by an increase in Treg-mediated immune tolerance mechanisms, and by impairment of the later stages of CD8 differentiation involved in the most potent antiviral activity [93,103]. Interestingly, these toxicities seem more frequent in treatment-naïve than in pre-treated patients and in younger than older individuals, further suggesting that the presence of a still partially preserved immune system is implied [101,103,104], and they might be associated with higher clinical activity [105].

Although the reduction of Tregs in the TME is an important and attractive therapeutic target, the caveat is that a reduction of Tregs activity, can activate autoimmune reactions [116,117]. For example, the effect on T-cells is believed to cause the severe diarrhea or colitis, which are some of the major side effects in patients receiving PI3Kδ inhibitors [7,102]. A picture similar to graft versus host disease has been described in these patients, with increase infiltration of CD8+ cytotoxic T-cells [118,119], perhaps due to the already mentioned effect of the PI3Kδ inhibitor on the mesenteric B-cells leading to an unleashed activity of Tregs [116].

We have also to consider that the pattern of selectivity for the PI3Kδ isoform versus other class IA or IB members largely varies across the small molecules that have entered the clinical evaluation (Table 1). Their ability to bind isoforms can affect the toxicity profile. An example is given by observed acute insulin resistance, also causing severe hyperglycemia and hyperinsulinemia, seen with compounds that also target PI3Kα, physiologically involved in the glucose homeostasis in muscle, liver, and fat tissues [6,102,120].

## 5. Effects on T-Cells in the Context of Clinical Trials

While the effect of the PI3Kδ inhibitors on the secretion of chemokines has been studied in many clinical trials enrolling patients with lymphoma (Table 3), only a few studies have explored whether the drugs affect T-cell populations in the peripheral blood (Pb) [114,117,121,122,123] or in the TME [108,124].

No significant changes in Pb T-cells subsets were seen in the phase I and II studies evaluating idelalisib in patients with relapsed indolent lymphoma [122,123] and in the phase I for CLL [114]. Conversely, a decrease of the Treg percentage was described in the Pb of 13/19 relapsed/refractory CLL patients treated for one month of idelalisib in a separate phase I study evaluating the small molecule as single agent followed by 6 months of combination therapy with the anti-CD20 antibody ofatumumab [117]. Importantly, the decrease of Tregs in the Pb was stronger in patients that experienced toxicity [117].

A reduction in the Pb Tregs was also observed in 14/19 relapsed/refractory CLL patients exposed to the PI3Kδ inhibitor ACP-319 in the phase I study [121].

Serial biopsies were obtained in 30 patients with relapsed/refractory solid tumors or lymphoma enrolled in a phase I study of copanlisib, a pan PI3K inhibitor, preferentially targeting the PI3Kα/PI3Kδ isoforms [108]. There was a reduction in the proportion of CD4+T-cells in tumors after 14 days of treatment in 14 of patients treated at 0.8 mg/Kg but not at 0.4 mg mg/Kg (*n* = 16), with no changes in the CD8+ cells [108]. The reduction in the CD4+ cells suggests that Tregs were affected; however no additional staining was performed.

In the phase I study, exploring the dual PI3K/BRAF inhibitor sonolisib in patients with advanced solid tumors bearing the *BRAF* V600 mutation, biopsies were performed at baseline and at day 8 of the first cycle in six patients [124]. An increase in CD8+ cells at immunohistochemistry was observed in 5/6 patients [124]. This was paired with higher PD-L1 staining in the two cases with a partial response and not in patients with stable or progressive disease [124]. Additionally, here, no data are available for Tregs.

Finally, since PI3Kδ is also downstream to FcεRI, activated by IgE binding in mast cells and basophils, idelalisib has been evaluated in patients with allergic rhinitis [125]. In a phase 1 study the PI3Kδ inhibitor decreased plasma levels of CD631/CCR31 basophils, and inhibited ex vivo basophil activation in response to allergen stimulation [125]. A similar effect has also been reported in relapsed/refractory lymphoma patients enrolled in a phase I with the PI3Kδ inhibitor dezapelisib [126].

## 6. Conclusions

PI3Kδ inhibitors are active anti-cancer compounds in lymphomas. Their mechanism of action is promiscuous, and it is mediated via a direct inhibition of PI3Kδ in the lymphoma cells but also due to an inhibitory activity in multiple non-neoplastic cells. In particular, the data we have summarized highlight that the pharmacological inhibition of PI3Kδ in Tregs is clearly effective in boosting anti-tumor immune system. Further studies are needed to exploit this therapeutic option, avoiding the possible insurgence of autoimmune disorders. Discovery of other pathways and molecules that preferentially inhibit PI3K signaling specifically in Tregs is needed.

## Figures and Tables

**Table 1:** List of P3Kdelta inhibitors sorted by their target, their official name, if assigned, or by their common/alternative name.

Target	Official Name	Common/Alternative Name	PI3Kδ (IC_50_, nM)	PI3Kα (IC_50_, nM)	PI3Kβ (IC_50_, nM)	PI3Kγ (IC_50_, nM)	Adm. Route	Phase ^#^	FDA Approval	On-Going Trials ^##^
PI3Kδ	Acalisib	*GS-9820, CAL-120* [18]	12.7	5441	3377	1389	p.o	1	-	-
PI3Kδ	Dezapelisib	INCB040093 [19]	31	28,912	3751	2297	p.o	2	-	-
PI3Kδ	Idelalisib	CAL-101, GS-1101 [20]	2.5	820	565	89	p.o	3	CLL, FL, SLL **	Lymphoid tumors
PI3Kδ	Leniolisib	*CDZ173* [21]	1.1	244	424	2230	p.o	3 ^^^	-	APDS/PASLI ^^^
PI3Kδ	Linperlisib	YY-20394, PI3K(delta)-IN-2 [22]	n.a.	n.a.	n.a.	n.a.	p.o	2	-	Lymphoid and solid tumors
PI3Kδ	Nemiralisib	*GSK2269557* [23]	9.9	n.a.	n.a.	n.a.	inh.	2^^^	-	-
PI3Kδ	Parsaclisib	INCB050465, *IBI-376* [19]	1.1	>20,000	>20,000	>10,000	p.o	3	-	Lymphoid tumors, myeloid neoplasms
PI3Kδ	Puquitinib	XC-302 [24]	3.3	992.8	959.2	89.8	p.o	no	-	-
PI3Kδ	Seletalisib	UCB-5857 [25]	12	3638	2129	282	p.o	2	-	No
PI3Kδ	Zandelisib	ME-401, PWT143 [26]	5	5022	208	2137	p.o	2	-	Lymphoid tumors
PI3Kδ	-	ACP-319, AMG 319 [27]	18	33,000	270	85	p.o	2	-	Lymphoid tumors
PI3Kδ	-	BGB-10188 [28]	n.a.	n.a.	n.a.	n.a.	p.o	2	-	Lymphoid and solid tumors
PI3Kδ	-	GS-9901 [29]	1	750	100	190	p.o	1	-	-
PI3Kδ	-	*GSK2292767* [23]	n.a.	n.a.	n.a.	n.a.	inh.	1 ^^^	-	-
PI3Kδ	-	HMPL-689 [30]	0.8	>1000	87	114	p.o	1	-	Lymphoid tumors
PI3Kδ	-	IOA-244, MSC2360844 [31]	145	18,500	2850	>20,000	p.o	1	-	Lymphoid and solid tumors
PI3Kδ	-	RV1729 [32]	12	193	n.a.	25	inh.	1 ^^^	-	-
PI3Kδ	-	*SHC014748M* [33]	n.a.	n.a.	n.a.	n.a.	p.o	2	-	Lymphoid tumors
PI3Kα/PI3Kδ	Copanlisib	BAY 80-6946 [34]	0.7	0.5	3.7	6.4	i.v.	3	FL ***	Lymphoid and solid tumors
PI3Kα/PI3Kδ	Pictrelisib	Pictilisib *GDC-0941, RG-7321* [35]	3	3	33	75	p.o	2	-	-
PI3Kα/PI3Kδ	-	*TQ-B3525* [36]	n.a.	n.a.	n.a.	n.a.	p.o	2	-	Lymphoid and solid tumors
PI3Kβ/PI3Kδ	-	*AZD8186* [37]	12	35	4	675	p.o	2	-	Solid tumors
PI3Kβ/PI3Kδ	-	KA2237 [38]	8	>500	19	>500	p.o	1	-	-
PI3Kα/PI3Kδ/PI3Kγ	Taselisib	*GDC-0032* [39]	0.12	0.29	9.1	0.97	p.o	3	-	Lymphoid and solid tumors
PI3Kα/PI3Kβ/PI3Kδ	Sonolisib	*PX-866* [40]	2.7	5.5	>300	9	p.o	2	-	-
PI3Kδ/PI3Kγ	Duvelisib	*IPI-145, INK1197* [41]	2.5	1602	85	27	p.o	3	CLL, FL, SLL ****	Lymphoid tumors
PI3Kδ/PI3Kγ	Tenalisib	RP6530 [42]	24	>7000	>3000	33	p.o	2	-	Solid tumors
PI3Kα/PI3Kδ/BRAF	-	*ASN003* [43]	6	16	690	97	p.o	1	-	-
PI3Kδ/CK1ε	*Umbralisib*	TGR-1202, RP5264 [44]	22.23	>9000	>1000	>1000	p.o	3	FL, MZL *****	Lymphoid tumors
PI3Kα/PI3Kβ/PI3Kδ/HDAC	*Fimepinostat*	CUDC-907 [45]	39	19	54	311	p.o	2	-	Lymphoid and solid tumors

FDA, U.S. Food and Drug Administration; target IC_50_ inhibition based on reported kinase inhibition profiles; ^#^, based on http://adisinsight.springer.com/ and on https://clinicaltrials.gov accessed in 15 September 2021; ^##^, defined as “recruiting” or “not yet recruiting” in https://clinicaltrials.gov accessed in 15 September 2021; APDS/PASLI, Activated phosphoinositide 3-kinase delta syndrome/p110δ-activating mutation causing senescent T-cells, lymphadenopathy and immunodeficiency; CLL, chronic lymphocytic leukemia; SLL, small lymphocytic lymphoma; FL, follicular lymphoma; MZL, marginal zone lymphoma; ^^^, non in oncology; **, for the treatment of patients with (a) relapsed CLL in combination with rituximab, in patients for whom rituximab alone would be considered appropriate therapy due to other co-morbidities, (b) relapsed FL after at least two prior systemic therapies, (c) relapsed SLL after at least two prior systemic therapies) [46]; ***, for the treatment of adult patients with relapsed FL after at least two prior systemic therapies [47]; **** for the treatment of adult patients with (a) relapsed or refractory CLL/SLL after at least two prior therapies, (b) relapsed or refractory FL after at least two prior systemic therapies [48]; ***** for the treatment of adult patients with (a) relapsed or refractory MZL who have received at least one prior anti-CD20-based regimen and (b) for relapsed or refractory FL who have received at least three prior lines of systemic therapy [49]; inh., inhalation.; p.o., per os; i.v., intravenous.

**Table 2:** Effect of the PI3Kδ inhibitors on serum levels of secreted factors in the context of clinical trials enrolling patients with lymphoma.

PI3Kδ Inhibitor	Phase	Lymphoma Subtypes	Decreased Factors	Increased Factors
Copanlisib	1 [108]	FL, WM, DLBCL, BL, MCL, PTCL	CCL2, CCL3, CCL5, CCL15, CCL16, IL-10, IL2RA, CD27, CD5L (cycle 1, day 15)	-
Duvelisib	1 [109]	FL, WM, SLL, MZL	CCL1, CCL4, CCL17, CCL22, CXCL10, CXCL13, IL-10, IL-16, MMP-9, TNFα (cycle 1, day 8)	-
Duvelisib	1 [110]	CLL	CCL1, CCL3, CCL4, CCL17, CCL22, CXCL10, CXCL13, IL-6, IL-10, IL-12p40, MMP-9, MMP-12, TNFα (cycle 1, day 8)	-
Duvelisib	1 [111]	PTCL	IL10, IL-12p40, CXCL13, (cycle 1, day 8)	CCL1, IL6, IL8, IL9, IL15 IL17A, IL-12p70, CD40L, TNFβ
Duvelisib	1 [112]	CLL, FL, WM, SLL, MZL	CCL1, CCL4, CCL17, CCL22, CXCL10, CXCL13, MMP-9, TNFα (cycle 1, day 8)	-
Duvelisib	3 [113]	CLL/SLL	CCL3, CCL4, CCL17, CCL19, CCL22, CXCL13, IL2RA, IL-12p40, IL-10, TNFα (cycle 2, day1);	-
Idelalisib	1 [114]	CLL/SLL	CCL3, CCL4, CCL17, CCL22, CD40L, CCL2, CXCL13, TNFα (within 1 month)	-
Tenalisib	1 [115]	HL	CCL17	-

FL, follicular lymphoma; WM, Waldenström’s macroglobulinemia; DLBCL, diffuse large B-cell lymphoma; BL, Burkitt lymphoma; MCL, mantle cell lymphoma; PTCL, peripheral T-cell lymphoma; SLL, small lymphocytic lymphoma; MZL, marginal zone lymphoma; CLL, chronic lymphocytic leukemia; HL, Hodgkin lymphoma.

**Table 3:** Potential toxicities of the PI3Kδ inhibitors in the context of clinical trials enrolling patients with lymphoma.

PI3Kδ Inhibitor	Phase	Lymphoma Subtypes	Any Grade, AE (%)	Grade ≥ 3, AE (%)
Copanlisib	2 [108]	FL, MZL, SLL, WM/LPL, DLBCL	Diarrhea (35.2), colitis (0.7), hyperglycemia (50.0), hypertension (29.6), neutropenia (28.9), pneumonitis (6.3)	Diarrhea (8.5), colitis (0.7), hyperglycemia (40.1), hypertension (23.9), neutropenia (24.0), pneumonitis (1.4)
Umbralisib	2 [109]	MZL, FL, SLL	Neutropenia (15.9), diarrhea (59.1), colitis (1.9), fatigue (30.8), increased ALT (20.2), increased AST (18.8)	Neutropenia (11.5), diarrhea (10.1), colitis (0.5), fatigue (3.4), increased ALT (6.7), increased AST (7.2)
Duvelisib	2 [113]	SLL, FL, MZL	Diarrhea (48.8), neutropenia (28.7), throbocytopenia (18.6), anemia (26.4), febrile neutropenia (9.3), increased ALT (14.0), increased lipase (9.3), pneumonia (7.8), colitis (7.8)	Diarrhea (14.7), neutropenia (24.8), throbocytopenia (11.6), anemia (14.7), febrile neutropenia (9.3), increased ALT (5.4), increased lipase (7.0), pneumonia (5.4), colitis (5.4)
Idelalisib	2 [114]	FL, SLL, MZL, WM/LPL	Diarrhea (43.0), pneumonia (11.0), increased ALT (47.0), increased AST (35.0)	Diarrhea (13.0), pneumonia (7.0), increased ALT (13.0), increased AST (8.0)
Tenalisib	1 [115]	DLBCL, MCL, PTCL, CLL, HL	Anemia (29.0), neutropenia (20.0), thrombocytopenia (26.0), pyrexia (37.0), cough (43.0), dyspnea (26.0)	Anemia (11.0), neutropenia (17.0), thrombocytopenia (17.0), pyrexia (3.0)

AE, adverse event; ALT, alanine aminotransferase; AST, aspartate eminotransferase; BL, Burkitt lymphoma; CLL, chronic lymphocytic leukemia; DLBCL, diffuse large B-cell lymphoma; FL, follicular lymphoma; LPL, Lymphoplasmacytic lymphoma; MCL, mantle cell lymphoma; MZL, marginal zone lymphoma; PTCL, peripheral T-cell lymphoma; SLL, small lymphocytic lymphoma; HL, Hodgkin lymphoma. WM, Waldenström’s macroglobulinemia.

## Abbreviations

APC	antigen presenting cells
CLL	chronic lymphocytic leukemia
CTL	cytotoxic T-lymphocyte
CTLA-4	Cytotoxic T-Lymphocyte Associated Protein 4
DLBCL	diffuse large B-cell lymphoma
ECM	extracellular matrix
NK	natural killer
PD-1	Programmed Cell Death 1
PI3K	phosphoinositide 3-kinases
TAM	tumor-associated macrophages
Tconvs	conventional T-cells
TFH	T follicular helper cells
TME	tumor microenvironment
Tregs	regulatory CD4+ T-cells
